# Analysis of Genetic Variation in CYP450 Genes for Clinical Implementation

**DOI:** 10.1371/journal.pone.0169233

**Published:** 2017-01-03

**Authors:** Liuh Ling Goh, Chia Wei Lim, Wey Cheng Sim, Li Xian Toh, Khai Pang Leong

**Affiliations:** 1 TTSH Research Laboratory, Clinical Research & Innovation Office, Tan Tock Seng Hospital, Singapore, Singapore; 2 Department of Rheumatology, Allergy and Immunology, Tan Tock Seng Hospital, Singapore, Singapore; University of South Alabama Mitchell Cancer Institute, UNITED STATES

## Abstract

**Background:**

Genetic determinants of drug response remain stable throughout life and offer great promise to patient-tailored drug therapy. The adoption of pharmacogenetic (PGx) testing in patient care requires accurate, cost effective and rapid genotyping with clear guidance on the use of the results. Hence, we evaluated a 32 SNPs panel for implementing PGx testing in clinical laboratories.

**Methods:**

We designed a 32-SNP panel for PGx testing in clinical laboratories. The variants were selected using the clinical annotations of the Pharmacogenomics Knowledgebase (PharmGKB) and include polymorphisms of CYP2C9, CYP2C19, CYP2D6, CYP3A5 and VKORC1 genes. The CYP2D6 gene allele quantification was determined simultaneously with TaqMan copy number assays targeting intron 2 and exon 9 regions. The genotyping results showed high call rate accuracy according to concordance with genotypes identified by independent analyses on Sequenome massarray and droplet digital PCR. Furthermore, 506 genomic samples across three major ethnic groups of Singapore (Malay, Indian and Chinese) were analysed on our workflow.

**Results:**

We found that 98% of our study subjects carry one or more CPIC actionable variants. The major alleles detected include CYP2C9*3, CYP2C19*2, CYP2D6*10, CYP2D6*36, CYP2D6*41, CYP3A5*3 and VKORC1*2. These translate into a high percentage of intermediate (IM) and poor metabolizer (PM) phenotypes for these genes in our population.

**Conclusion:**

Genotyping may be useful to identify patients who are prone to drug toxicity with standard doses of drug therapy in our population. The simplicity and robustness of this PGx panel is highly suitable for use in a clinical laboratory.

## Introduction

There is significant variability in drug response with regard to efficacy, optimal dose and adverse drug reactions (ADRs) [1–3]. Genetic variations have been estimated to contribute between 20–30% to variability in response to drugs [4]. Implementing PGx in routine clinical care has the potential to reduce adverse drug reactions and maximise drug efficacy based on the genetic profile of individual patients [5]. Approximately 18% of prescribed drugs carry actionable pharmacogenetic labels in the USA, demonstrating the increasing importance of genetic testing for clinical practice [6]. The incorporation of PGx into clinical practice requires accurate genotyping and correct prediction of genetic variants contributing to inter-individual differences in drug response.

The cytochrome P450 (CYP) enzymes are the major system that catalyse phase I drug metabolism, encompassing most of the clinically prescribed drugs [7]. The activity of each enzyme encoded by the combination of CYP450 star alleles is categorized as poor metabolizer (PM), intermediate metabolizer (IM), extensive metabolizer (EM) and ultra-rapid metabolizer (UM). Some of these genes are highly polymorphic with multiple alleles and the prediction of phenotypes by detecting polymorphisms of CYP genes that are important for drug metabolism is instrumental in drug therapy [8]. The genetic polymorphisms affecting activities of clinically relevant drug-metabolizing enzymes are found in CYP2C9, CYP2C19, CYP3A5 and CYP2D6. Genotyping CYP2C9 and VKORC1 aid in the management of patients receiving warfarin therapy [9,10]. CYP2C19 is important for the metabolism of a number of drugs including clopidogrel, omeprazole and phenytoin [11–13]. CYP3A5 is associated with the metabolism of tacrolimus [14]. Although CYP3A4 has overlapping substrate specificity as CYP3A5, no functional variant forms of CYP3A4 have been observed in Asians and Caucasians [15,16]. CYP2D6 is estimated to metabolize approximately 25% of all clinically used drugs, including codeine, fluoxetine and amitriptyline [17–19].

CYP2D6 is highly polymorphic and represents one of the most difficult enzymes to genotype. It spans a 4.3 Kb region on chromosome 22q13.1 and forms a cluster with two pseudogenes, CYP2D7 and CYP2D8 [20]. At present, more than 100 allelic variants and sub variants have been described [21]. Its genetic complexities are due to SNPs, duplications and multiplications, deletions and recombination events with the highly homologous CYP2D7 pseudogene. The frequency of CYP2D6 gene multiplication is as high as 45% in Asian [22]. It is important to accurately measure the exact copy number of functional CYP2D6 genes as not all copy number variations (CNVs) are functionally similar. The presence of different genetic variations mandates the use of more than one assay to determine the CYP2D6 genotypes. For example, the CYP2D6-CYP2D7 fusion genes, such as CYP2D6*36, cannot be discriminated from CYP2D6*10 that shares the same SNP (100C>T) without the identification of the sequence of each copy. Hence, it is important to identify which of the two alleles carries the duplication or multiplication in order to predict the phenotype accurately in a clinical setting. This piece of information serves as a guide for individualization of drug therapy.

The pharmacogenetic testing of CYP polymorphisms in a clinical laboratory needs to be rapid, robust and cost-effective. Available methods for CYP genotyping include restriction fragment length polymorphism analysis, allele-specific PCR or Sanger sequencing which are time consuming. Some of the available commercial kits are limited in coverage, omitting significant variations relevant for Asian populations. In this study, we explored the use of a 32-SNP panel with TaqMan genotyping assays coated in 384-well plate format and 2 copy number assays for the detection of significant variations in CYP2C9, CYP2C19, CYP3A5 and CYP2D6. We genotyped 506 subjects from our population and defined the allele frequencies and phenotypic consequences in different ethnic groups. The results are used to assess the associations of pharmacogenetics and drug response; and to assess the feasibility of implementing PGx testing in different ethnic groups. Our study contributed to the overall knowledge of the Singapore prevalence of CYP450 genotypes. Alongside with the simplicity and specificity of the TaqMan genotyping assays, this test is highly applicable in a clinical laboratory.

## Methods

### Sample collection and processing

In this study, we used 10 HapMap samples for assay validation. All HapMap samples were purchased from Coriell Cell Biorepositories. We tested 506 healthy individual samples from the three major ethnic groups (126 Malays, 179 Indians and 201 Chinese) in Singapore provided by TTSH BioBank. All subjects provided written consent. The study was approved by the institutional ethics review board. DNA was extracted from peripheral blood collected with EDTA blood tubes using the QIAamp^®^ DNA Blood Mini QIAcube Kit (QIAGEN, P/N: 51126). The automated process was performed on the QIAcube^®^ system according to manufacturer’s recommended protocols. Extracted DNA samples were quantified by NanoDrop 2000c Spectrophotometer and normalised to 5ng/μl for use in genotyping and copy number variation experiments.

### 32 SNP genotyping experiments

Genotyping was performed using TaqMan-based real-time PCR with TaqMan assays from Applied Biosystems^™^ (Table 1). Pre-coated 384-well TaqMan^®^ Custom Plating (Applied Biosystems^™^, P/N: 4462783) which contains 32 pharmacogenomics assays was used. Up to 11 samples plus one no-template control were included in each run. A final volume of 5 μl consisting of 2.5 μl TaqMan^®^ Genotyping Master Mix (Applied Biosystems^™^, P/N: 4371355) and 2.5 μl genomic DNA was dispensed to each well by epMotion M5073 automated pipetting system (Eppendorf). The TaqMan^®^ Genotyping Master Mix (Applied Biosystems^™^, P/N: 4371355) contains ROX dye which serves as a passive reference dye to normalize signal and ensure data integrity. The qPCR was run on QuantStudio^™^ 6 Flex Real-Time PCR System (Applied Biosystems^™^) with the following thermal cycling condition: pre-PCR hold at 60°C for 30 seconds, initiation at 95°C for 10 minutes for initial denaturation and enzyme activation, followed by 50 cycles of 95°C for 15 seconds and 60°C for 90 seconds.

**Table 1:** Loci and alleles detected by the assays.

Gene	Allele	dbSNP Number	Major Nucleotide Variation	Effect	Enzyme Activity
CYP2C9	*2	rs1799853	c. 430C>T	R144C	Decreased
	*3	rs1057910	c. 1075A>C	I359L	Decreased
	*4	rs56165452	c. 1076T>C	I359T	Decreased
	*5	rs28371686	c. 1080C>G	D360E	Decreased
	*6	rs9332131	c. 818delA	273 Frameshift	None
CYP2C19	*2	rs4244285	c. 681G>A	Splicing defect	None
	*3	rs4986893	c. 636G>A	W212X	None
	*4	rs28399504	c. 1A>G	M1V	None
	*5	rs56337013	c. 1297C>T	R433W	None
	*6	rs72552267	c. 395G>A	R132Q	None
	*7	rs72558186	c. 819+2T>A	Splicing defect	None
	*8	rs41291556	c. 358T>C	W120R	None
	*9	rs28399507	c. 431G>A	R144H	Decreased
	*10	rs6413438	c. 680C>T	P227L	Decreased
	*17	rs12248560	c. -806C>T	Increased expression	Increased
CYP2D6	*2	rs16947, rs1135840	2850C>T, 4180G>C	R296C, S486T	Normal
	*2A	rs1080985, rs16947, rs1135840	-1584C>G, 2850C>T, 4180G>C	R296C, S486T	Normal
	*3	rs35742686	2549delA	259 Frameshift	None
	*4	rs1065852, rs3892097, rs1135840	100C>T, 1846G>A, 4180G>C	P34S, Splicing defect, S486T	None
	*5	n/a	n/a	Gene deletion	None
	*6	rs5030655	1707delT	118 Frameshift	None
	*7	rs5030867	2935A>C	H324P	None
	*8	rs5030865, rs16947, rs1135840	1758G>T, 2850C>T, 4180G>C	G169X, R296C, S486T	None
	*9	rs5030656	2615_2617delAAG	K281del	Decreased
	*10	rs1065852, rs1135840	100C>T, 4180G>C	P34S, S486T	Decreased
	*14	rs5030865, rs16947, rs1135840	1758G>A, 2850C>T, 4180G>C	G169R, R296C, S486T	None
	*17	rs28371706, rs16947, rs1135840	1023C>T, 2850C>T, 4180G>C	T107I, R296C, S486T	Decreased
	*29	rs16947, rs59421388, rs1135840	2850C>T, 3183G>A, 4180G>C	R296C, V338M, S486T	Decreased
	*35	rs769258, rs16947, rs1135840	31G>A, 2850C>T, 4180G>C	V11M, R296C, S486T	Normal
	*36	rs1065852, rs1135840	100C>T, 4180G>C, recombination at exon 9	P34S, S486T, CYP2D6-2D7 hybrid	None
	*41	rs16947, rs28371725, rs1135840	2850C>T, 2988G>A, 4180G>C	R296C, Splicing defect, S486T	Decreased
CYP3A5	*3	rs776746	6986A>G	Splicing defect	None
VKORC1	*2	rs9923231	c. -1639G>A		Decreased

### CYP2D6 copy number variation analysis

CYP2D6 gene copy number was determined using Applied Biosystems’ commercially available TaqMan Copy Number Assays, Exon 9 (Assay ID: Hs00010001_cn) and Intron 2 (Assay ID: Hs04083572_cn). Exon 9 copy number assay was used to quantify all non-CYP2D6*36 alleles and identify CYP2D6 gene deletion (*5) in the samples. Intron 2 copy number assay was used to quantify all alleles including CYP2D6*36 alleles. The total copy number variation difference between the two copy number assays were used to identify the actual copy number of CYP2D6*10 and CYP2D6*36 alleles. All assays were run together with a reference gene, the commercially available TaqMan Copy Number Reference Assay RNaseP (Assay ID: 4403326, Applied Biosystems^™^). All samples were prepared in quadruplicates in a 384-well format with the TaqMan Genotyping Master Mix (Applied Biosystems^™^, P/N: 4371355) using 10ng of genomic DNA in a 10 μl reaction. The qPCR was carried out using the QuantStudio^™^ 6 Flex Real-Time PCR System with the following thermal cycling conditions: initiation at 95°C for 10 minutes for initial denaturation and enzyme activation, followed by 40 cycles of 95°C for 15 seconds and 60°C for 1 minute.

### Data analysis

The TaqMan genotyping analysis was performed according to the manufacturer's recommendations and published protocol. The run (.eds) files were first analysed using the QuantStudio^™^ Real-Time PCR Software v1.2 to check for the overall quality of the amplification of all 32 assays. The end-point data files were then imported into Applied Biosystems^®^ TaqMan^®^ Genotyper Software v1.3.1 to verify the genotype calls and clustering patterns. The analysis was done by Autocalling with pre-defined algorithmic approach and information from imported Assay Information File (AIF). Manual calling was done to overwrite the auto-calling if necessary. The analysed genotype results were exported into a tab-delimited (.txt) file using the “Advanced’ settings before being incorporated into AlleleTyper^™^ Software v1.0.7-I0B01 (Applied Biosystems^™^) to determine the haplotypes.

For CYP2D6 gene copy number analysis, relative quantitation (RQ) analysis was performed using CopyCaller^™^ Software (Applied Biosystems^™^). The raw results were first analysed using the QuantStudio^™^ Real-Time PCR Software v1.2 with a manual C_T_ threshold level of 0.2 and auto baseline settings to determine C_T_ values. The results were exported to tab-delimited (.txt) files, before being imported into the CopyCaller^®^ Software to determine the copy number using the comparative C_T_ (ΔΔC_T_) method. The HapMap DNA sample, NA18558 (Coriell Institute), was used as the calibrator sample, with a known copy number value of 2 for both Exon 9 and Intron 2 assays. Copy number results were then exported to tab-delimited (.txt) files to be used in the AlleleTyper^™^ Software.

The results of the genotype calls and the copy number variations were incorporated into AlleleTyper^™^ Software v1.0.7-I0B01 to determine the haplotypes of the samples. The star-allele calls were performed based on the pre-set translation tables.

### Phenotype translation

The genotyping data were used to assign PGx phenotypes based on current understanding of the roles of these genes in drug metabolism and the functional consequences of the identified sequence alterations. Star allele assignments and genotype to phenotype correlation were adapted from information available at the human Cytochrome P450 Allele Nomenclature Committee website (http://www.cypalleles.ki.se). The phenotype algorithms were derived from the Clinical Pharmacogenetics Implementation Consortium (CPIC) guidelines at Pharmacogenomics Knowledgebase (PharmGKB) website (https://www.pharmgkb.org). The method used to predict drug metabolizer phenotype for CYP2D6 included intermediate phenotype categories (EM to UM, IM to EM and PM to IM) according to a recent paper and was summarized in S1 Fig. [23].

### Alternative molecular tests for validation

Ten HapMap samples were tested on an alternative platform to verify our genotype calls and copy number variation results. Results of the 28 SNPs or INDEL assays and CYP2D6 intron 2 copy number assay in our panel were compared with the results interrogated by iPLEX^®^ PGx 68 Panel (Agena Bioscience) using MALDI-TOF mass spectrometry. Multiplex PCR was performed followed by dephosphorylation with alkaline phosphatase and primer extension reactions. The products were spotted onto MassArray SpectroCHIP and analysed using the MassARRAY^®^ system. The concordance of our CYP2D6 exon 9 copy number assay results were validated by running the 10 HapMap samples on QX200^™^ Droplet Digital^™^ PCR (ddPCR) System (Bio-Rad Laboratories Inc, P/N: 1864001). In this system, PCR reaction occurs independently in individual droplets, which are subjected to fluorescence reading after amplification. The same Taqman probe was used according to published protocol [24].

### Statistical analysis

The chi-squared test with one degree of freedom was used to test the departure from Hardy-Weinberg equilibrium (HWE) for each variant allele in different ethnic group. All the genotypes were in Hardy-Weinberg Equilibrium (HWE). Wright’s fixation index (*F*_st_) was calculated to assess the variance in SNPs across the three ethnicities (Malay, Indian, and Chinese) in the Singapore population. *F*_st_ values range from 0 to 1; values less than 0.05 indicate little differentiation and those greater than 0.15 show substantial differentiation.

## Results

### Assay verification

The major genetic polymorphisms of CYPP450s drug metabolism are caused by variations in *CYP2D6*, *CYP2C9*, *CYP2C19* and *CYP3A5* genes. A customized panel that allows for detection of 32 gene variations from a single run on 384-well plate format was evaluated for clinical use (Table 1). Key variants of CYP2C9, CYP2C19, CYP2D6, CYP3A5 and VKORC1 were interrogated by TaqMan assays. These 32 variants were identified from literature, CYP allele website and selected based on the curated pharmacogenetics data and PharmGKB. Their clinical evidence and prevalence in Asians are the basis of selection. DNA samples from 10 Coriell Cell Repository with known genotypes in rs1057910, rs28399504, rs776746 and rs9923231 were used to assess the performance of the genotyping assays. The assays performed well with 31 assays having unambiguous genotypes, yielding 96.8% call rate. Only CYP2C9*6 required manual calling due to high background of the VIC-dye labelled Taqman probe (data not shown). The haplotypes of the Coriell samples were shown in Table 2. As most of the alleles examined in this study were absent in these samples, an alternative test method based on MALDI-TOF mass spectrometry was included for validation. The PGx68 panel (Agena Biosciences) was used to analyse 28 alleles in our current study. The 10 Coriell samples were run in duplicates and the results were in concordance with the known genotypes and between the 2 platforms (S2 Fig). There were 4 alleles that were interrogated by only one assay and have not been confirmed by a separate method. These included rs28399507 and rs6413438 of CYP2C19 and rs1080985 and rs769258 of CYP2D6. We compared their allele frequencies with those in ExAC database and published literatures. The similar allelic frequencies suggested that our assays for these variants were valid.

**Table 2:** Haplotype calls of 10 HapMap samples used for assay validation.

No.	Sample ID	Sample Name	Exon 9	Intron 2	CYP2D6 without CN	CYP2D6 with CN	CYP2C19	CYP2C9	VKORC1	CYP3A5
1	H01	NA18592	2	3	*2A/*10	*2AX2/*36	*1/*1	*1/*1	*2/*2	*3/*3
2	H02	NA18532	2	4	*10/*10	(*10/*36)X2	*1/*1	*1/*1	*2/*2	*3/*3
3	H03	NA18526	2	4	*1/*10	(*1/*36)X2	*1/*1	*1/*1	*2/*2	*1/*1
4	H04	NA18610	2	4	*10/*10	(*10/*36)X2	*1/*3	*1/*1	*2/*2	*1/*3
5	H05	NA18568	2	3	*2A/*10	*2AX2/*36	*1/*1	*1/*1	*2/*2	*3/*3
6	H06	NA18595	2	3	*1/*10	*1X2/*36	*1/*2	*1/*1	*2/*2	*3/*3
7	H07	NA18524	2	4	*1/*10	(*1/*36)X2	*1/*2	*1/*3	*2/*2	*1/*3
8	H08	NA18548	2	4	*10/*10	(*10/*36)X2	*1/*1	*1/*1	*2/*2	*1/*1
9	H09	NA18534	2	2	*2A/*10	*2A/*10	*1/*2	*1/*1	*2/*2	*3/*3
10	H10	NA18558	2	2	*1/*10	*1/*10	*1/*2	*1/*1	*2/*2	*3/*3

CN, copy number.

The genotypes for CYP2D6 was determined with CNVs using 2 TaqMan assays targeting exon 9 and intron 2 [25]. As shown in Table 2, the use of two different assays enabled the differentiation of *36 from *10. The accuracy of exon 9 and intron 2 CNV assays were evaluated by alternate platforms using mass array and ddPCR, respectively. There was good concordance between our assays and the alternative assays (S3 Fig).

### Allele frequencies

The assays were applied to samples from 506 healthy Singapore individuals. The individual allele frequencies for the 5 genes were summarized in Table 3. The frequencies of the various alleles were highly similar between the Chinese and Malay ethnic groups. A greater differentiation was observed between the Indian and Chinese/Malay populations. Overall, the allelic frequencies were similar to those reported in the ExAC database (East Asian—Chinese and Malay; South Asian—Indian). Five different alleles of CYP2C9 were examined and the frequencies of the defective alleles *2 and *3 found in our study were comparable with published data [26]. The *3 allele was more common in our population whereas *2 allele is only observed in the Indians with a frequency of 0.042. The other deficient alleles for CYP2C9 (*4, *5 and *6) were not observed in the study population.

**Table 3:** Allele frequencies in Malay, Chinese and Indian subjects.

Gene	Common allele	Allele frequencies (this study)				Allele frequencies (ExAC)	
		Malay	Indian	Chinese	*F*st^a^	East Asian	South Asian
CYP2C9	*2	0.000	0.042	0.002	0.026	0.000	0.046
	*3	0.060	0.128	0.027	0.027	0.034	0.113
CYP2C19	*2	0.274	0.380	0.373	0.010	0.310	0.340
	*3	0.028	0.011	0.037	0.005	0.067	0.004
	*6	0.000	0.000	0.005	0.003	0.001	0.000
	*17	0.024	0.151	0.010	0.070	ND	ND
CYP2D6	*2 (rs16947)	0.143	0.341	0.137	0.055	0.852	0.620
	*2 (rs1135840)	0.655	0.489	0.706	0.036	0.299	0.481
	*2A	0.115	0.215	0.107	0.019	ND	ND
	*4	0.028	0.089	0.002	0.035	0.004	0.116
	*5	0.024	0.039	0.017	0.003	ND	ND
	*7	0.000	0.008	0.000	0.005	0.000	0.008
	*9	0.004	0.006	0.000	0.002	0.000	0.002
	*10	0.258	0.042	0.236	0.064	0.591	0.181
	*14	0.000	0.000	0.007	0.005	0.016	0.000
	*35	0.000	0.011	0.000	0.007	0.003	0.011
	*36	0.210	0.014	0.313	0.105	ND	ND
	*41	0.040	0.134	0.032	0.034	0.030	0.135
CYP3A5	*3	0.587	0.637	0.734	0.016	ND	ND
VKORC1	*2	0.710	0.182	0.898	0.382	ND	ND

ND, Not Determined.

For CYP2C19, *2 allele was the most common allele in our population, followed by *3 and *17 alleles [27,28]. The *3 allele frequency was higher in Chinese (0.037) and Malay (0.028) subjects, compared to the Indian subjects (0.011). By contrast, the *17 allele was more prevalent among the Indians subjects (0.151), compared to the Chinese (0.01) and Malay (0.024) subjects. The *6 allele was rare and the other deficient alleles for CYP2C19 (*4, *5, *7, *8, *9 and *10) were not observed in the study population.

The polymorphisms in CYP2D6 *2 (rs16947, rs1135840), *2A (rs1080985), *10 (rs1065852) and *41 (rs28371725) are common in our population [29]. The Chinese and Malay subjects exhibited approximately five-fold higher allele frequencies of *10 (rs1065852) compared with the Indian subjects. Similarly, the *2 (rs1135840) allele frequencies was approximately 2-fold higher in Chinese and Malay subjects than the Indians subjects. By contrast, the variant frequencies of *2 (rs16947), *2A (rs1080985), *4 (rs3892097) and *41 (rs28371725) were higher in the Indian subjects compared with the Chinese and Malay subjects. The *7 (rs5030867), *9 (rs5030656) and *35 (rs769258) alleles were rare and only observed in the Indian subjects. The *14 (rs5030865) allele was also rare and only observed in the Chinese subjects. The other deficient alleles, *3 (rs35742686), *6 (rs72552267), *8 (rs5030865), *17 (rs28371706) and *29 (rs59421388) were not detected in this study.

In line with previous findings, CYP3A5*3 is prevalent in our population with allele frequencies ranging from 0.587 to 0.734 [26]. VKORC1*2 differed by approximately 5 folds within our population. Notable difference was observed for rs9923231 with *Fst* 0.382. It was more common in the Chinese and Malay subjects than the Indian subjects.

### Diplotypes frequencies and phenotype consequences

**Fig 1:**
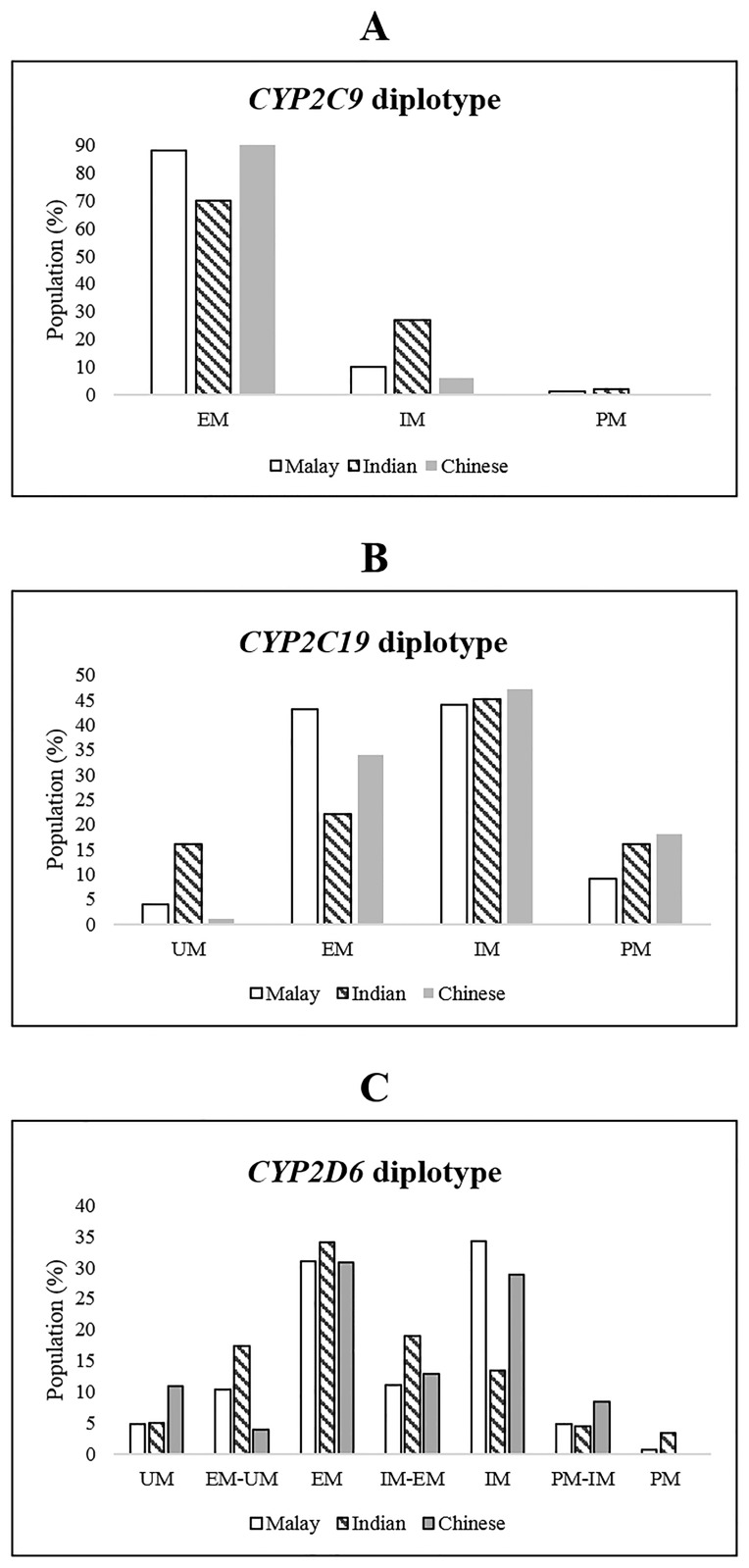
Frequency of metabolizer groups in 3 major ethnic groups of Singapore population. (A) CYP2C9 (B) CYP2C19 and (C) CYP2D6.

Table 4 shows the distribution of diplotypes among Chinese, Malay and Indian subjects. Phenotypes were inferred as described in S1 Fig and the frequencies of the various combinations of phenotype classes are depicted in Fig 1. CYP2C9*2 and *3 alleles are present in our population and both conferred reduced activity. The *3 allele is known to be prevalent in Asians and accounts for approximately 11.8% (60/506) of IM and 1% (5/506) of PM in our population. We noted a higher proportion of IM and PM among the Indian subjects, followed by the Malay subjects and the Chinese subjects (Fig 1A).

**Table 4:** Diplotype frequencies and functional classification of CYP2C9, CYP2C19, CYP2D6, CYP3A5 and VKORC1 in the Singapore population (n = 506).

Gene	Diplotype	Phenotype	Malay (n = 126)		Indian (n = 179)		Chinese (n = 201)	
			Total	Frequency	Total	Frequency	Total	Frequency
CYP2C9	*1/*1	EM	112	0.889	124	0.693	189	0.940
	*1/*2	IM	0	0.000	13	0.073	1	0.005
	*1/*3	IM	13	0.103	36	0.201	11	0.055
	*2/*3	PM	0	0.000	2	0.011	0	0.000
	*3/*3	PM	1	0.008	4	0.022	0	0.000
CYP2C19	*1/*1	EM	54	0.429	39	0.218	68	0.338
	*1/*2	IM	48	0.381	55	0.307	84	0.418
	*1/*3	IM	6	0.048	1	0.006	9	0.045
	*1/*5	IM	1	0.008	0	0.000	0	0.000
	*1/*8	IM	0	0.000	1	0.006	0	0.000
	*1/*17	UM	5	0.040	27	0.151	2	0.010
	*2/*2	PM	9	0.071	26	0.145	29	0.144
	*2/*3	PM	1	0.008	3	0.017	4	0.020
	*2/*5	PM	1	0.008	0	0.000	0	0.000
	*2/*6	PM	0	0.000	0	0.000	2	0.010
	*2/*17	IM	1	0.008	25	0.140	2	0.010
	*3/*3	PM	0	0.000	0	0.000	1	0.005
	*17/*17	UM	0	0.000	1	0.006	0	0.000
	UND		0	0.000	1	0.006	0	0.000
CYP2D6	*1/*1	EM	13	0.103	39	0.218	14	0.070
	*1/*35	EM	0	0.000	2	0.011	0	0.000
	*1X2/*36	EM	18	0.143	2	0.011	42	0.209
	(*1/*36)X2	EM	3	0.024	1	0.006	1	0.005
	*2A/*9	EM	0	0.000	2	0.011	0	0.000
	*2A/*10	EM	4	0.032	6	0.034	5	0.025
	*2A/*41	EM	1	0.008	9	0.050	0	0.000
	*1/*2A	EM-UM	13	0.103	31	0.173	8	0.040
	*1/*4	IM	2	0.016	13	0.073	1	0.005
	*1/*5	IM	3	0.024	4	0.022	1	0.005
	*1/*7	IM	0	0.000	2	0.011	0	0.000
	*4/*35	IM	0	0.000	1	0.006	0	0.000
	*4X2/*36	IM	1	0.008	0	0.000	0	0.000
	*9/*10	IM	1	0.008	0	0.000	0	0.000
	*10/*10	IM	10	0.079	0	0.000	2	0.010
	*10/*41	IM	3	0.024	2	0.011	3	0.015
	*10X2/*36	IM	14	0.111	1	0.006	21	0.104
	(*10/*36)X2	IM	7	0.056	0	0.000	23	0.114
	*10X2/*36X3	IM	0	0.000	0	0.000	2	0.010
	*10X2/*36XN	IM	0	0.000	0	0.000	1	0.005
	*41/*41	IM	0	0.000	1	0.006	0	0.000
	*36/*41X2	IM	2	0.016	0	0.000	3	0.015
	(*36/*41)X2	IM	0	0.000	0	0.000	1	0.005
	*1/*10	IM-EM	12	0.095	4	0.022	18	0.090
	*1/*41	IM-EM	1	0.008	25	0.140	6	0.030
	*2A/*4	IM-EM	1	0.008	2	0.011	0	0.000
	*2A/*5	IM-EM	0	0.000	3	0.017	1	0.005
	*10X3/*36	IM-EM	0	0.000	0	0.000	1	0.005
	*4/*4	PM	0	0.000	2	0.011	0	0.000
	*4/*5	PM	1	0.008	3	0.017	0	0.000
	*5/*5	PM	0	0.000	1	0.006	0	0.000
	*4/*10	PM-IM	0	0.000	2	0.011	0	0.000
	*4/*41	PM-IM	1	0.008	4	0.022	0	0.000
	*5/*10	PM-IM	1	0.008	0	0.000	5	0.025
	*5/*41	PM-IM	1	0.008	2	0.011	0	0.000
	*10/*36	PM-IM	2	0.016	0	0.000	8	0.040
	*10/*36X2	PM-IM	1	0.008	0	0.000	3	0.015
	*10/*36X3	PM-IM	0	0.000	0	0.000	1	0.005
	*1/*1X2	UM	0	0.000	2	0.011	1	0.005
	*1X3/*36	UM	1	0.008	0	0.000	2	0.010
	*2A/*2A	UM	1	0.008	5	0.028	2	0.010
	*2A/*2AX2	UM	0	0.000	1	0.006	0	0.000
	*2AX2/*36	UM	4	0.032	1	0.006	16	0.080
	(*2A/*36)X2	UM	0	0.000	0	0.000	1	0.005
	UND	UND	4	0.032	6	0.034	8	0.040
CYP3A5	*1/*1	EM	24	0.190	28	0.156	19	0.095
	*1/*3	IM	56	0.444	74	0.413	69	0.343
	*3/*3	PM	46	0.365	77	0.430	113	0.562
VKORC1	*1/*1		9	0.071	122	0.682	1	0.005
	*1/*2		55	0.437	49	0.274	39	0.194
	*2/*2		62	0.492	8	0.045	161	0.801

UM, ultra-rapid metabolizer; EM, extensive metabolizer; IM, intermediate metabolizer; PM, poor metabolizer; UND, undetermined

Among the CYP2C19 variants, *2 and *3 alleles are prevalent in our population. Both confer loss of function such that *1/2 and *1/3 heterozygous are assigned as IMs, and combinations of *2 and *3 alleles as PMs (Table 4). Due to the high frequency of *2 and *3 alleles in our population, there was subsequently a high proportion of IMs (about 45%). Fig 1B showed a higher proportion of PMs among the Indian and Chinese subjects (16%) compared to the Malay subjects (9%). In contrast, UMs were only prevalent among the Indian subjects (17%) and were less frequent among the Chinese (1%) and Malay (4%) subjects.

Several diplotypes observed in CYP2D6 (Table 4) were represented by 10 different alleles. Some alleles could not be unambiguously detected because their SNP combination did not match any known CYP2D6 alleles. The common alleles included *10, *36 and *41 alleles with reduced function that accounted for the IMs and PMs observed in our population. The gene allele quantification of CYP2D6 had to be determined simultaneously with its genotype in order to assign the correct phenotype. Some alleles (*1, *2 and *2A) were duplicated and accounted for the UMs in our study subjects. The frequencies of individuals with more than 2 copies ranged from 2.8% to 37%, based on the assays targeting exon 9 and intron 2, respectively (Table 5). The two copy number assays were concordant in only 62% of the 506 samples tested. This was not unanticipated as exon 9 assay did not detect CYP2D6*36 allele which was common among Asians while intron 2 assay detected all alleles. The use of these two assays targeting different regions of CYP2D6 helped to distinguish *10 and *36 alleles. The CYP2D6*36 allele is a gene conversion from CYP2D6 to CYP2D7 in exon 9. Detection of this allele was problematic and most commercial assays are not designed to detect *36, which is often misinterpreted as *10. The distinction of these 2 alleles is important as *36 is a non-functional allele whereas *10 is a decreased functional allele. This will have an impact on the risk of treatment failure or toxicity of drugs metabolized by CYP2D6. The observed diplotypes were classified into 6 phenotypes as depicted in Fig 1C. The diplotype frequencies predicting PM were the highest in the Indian subjects, followed by the Malay subjects and were absent in the Chinese subjects. Conversely, diplotypes predicting UM were highest in the Chinese, followed by the Indian and Malay subjects.

**Table 5:** Frequencies of CYP2D6 copy numbers in the Singapore population tested.

Copy number	Malay		Indian		Chinese		Total	
	Exon 9	Intron 2	Exon 9	Intron 2	Exon 9	Intron 2	Exon 9	Intron 2
0	0.000	0.000	0.006	0.006	0.000	0.000	0.002	0.002
1	0.071	0.048	0.073	0.067	0.104	0.035	0.085	0.049
2	0.897	0.516	0.888	0.860	0.876	0.368	0.885	0.579
3	0.032	0.349	0.034	0.061	0.020	0.433	0.028	0.281
4 and above	0.000	0.087	0.000	0.006	0.000	0.164	0.000	0.089

CYP3A5 and CYP3A4 have overlapping substrates specificity. We only included CYP3A5 in our panel as no functional variant forms of the CYP3A4 gene has been observed in Asians and Caucasian. A substantial proportion of CYP3A5 diplotypes in our population were *1/*3 and *3/*3 with intermediate and low activities, respectively (Table 4).

### Clinically relevant variants

**Fig 2:**
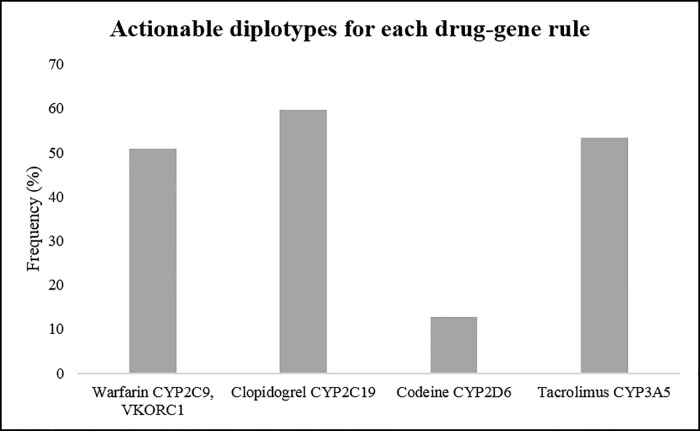
The frequencies of actionable diplotypes for each drug-gene rule. The definition of actionable diplotypes is defined as follows: (i) warfarin, CYP2C9 *2 or *3 heterozygote or homozygote with VKORC1 GA or AA genotype and CYP2C9*1 with VKORC1 AA genotype; (ii) clopidogrel, CYP2C19*2, *3 and *6; (iii) codeine, CYP2D6 poor and intermediate metabolizers; (iv) tacrolimus, CYP3A5*1 heterozygote or homozygote.

We determined the expected rates of actionable PGx variants in each CYP450 gene examined in our population. The proportions of individuals carrying an actionable variant were 14.2%, 16%, 67.8% and 84.4% in CYP3A5, CYP2C9, CYP2C19 and CYP2D6, respectively. Considering all four CYP450 genes together, 98% of the subjects carried an actionable PGx variant in at least one gene. We next determined the proportion of individuals carrying actionable genotypes for four drugs with evidence level 1A in CPIC (Fig 2 and Table 5). We noted a high proportion of individuals (approximately 50%) carrying actionable genotypes for warfarin and tacrolimus. It was noted that the CYP3A5*1 genotype codes for functional CYP3A5 enzyme but standard dosing recommendations for tacrolimus are based on individuals with the CYP3A5*3 (non-functional) genotype. Approximately 25% and 35% of individuals carried actionable genotypes for codeine and clopidogrel, respectively.

## Discussion

In this study, we developed an appropriate panel relevant to the prescription patterns and genotype prevalence in this country. Several factors, including cost, ease of workflow, turnaround time and the flexibility of the assay were considered in when choosing the genotyping approach. We built the panel utilizing the Life technologies TaqMan assays for both genotyping and copy number analyses. The panel in a 384-well format can detect 32 genetic variants in CYP450 genes simultaneously and CYP2D6 copy number assays were performed separately in the same platform. Data analysis tools were provided by Life technologies to facilitate the translation of individual’s genotypes into star allele haplotypes. To access the quality of the panel, we measured concordance of genotypes determined by independent platforms. The results showed a high call rate (96.8%) with 1 out of 32 SNPs requiring manual calling (CYP2C9*6) and 100% concordance for 28 SNPs with known genotypes or verified on alternative platform. To test the workflow for clinical use, we attempted to predict the phenotypes from the genotype data using guidelines from CPIC which provides information on allele frequencies and instructions to translate diplotypes into predicted phenotypes.

There is considerable diversity in the frequency of PGx variants that contribute to inter-population differences in drug response. Hence, 506 subjects from three distinct Asian ethnic groups in our population were analysed on our panel. Most of the data generated in this study corroborated data previously reported for the 3 major ethnic groups in our population. For example, CYP2C19*3, which is virtually absent in Caucasians, is common among East Asians [27]. Similarly, our subjects showed high prevalence of CYP2C19*2 and *3 loss of function alleles, implying that a large proportion of individuals in our population have poor response to clopidogrel; hence testing for CYP2C19 variants in our population may be useful. Clopidogrel has been identified as a high-priority drug for clinical implementation of PGx testing [30]. Individuals with reduced function phenotypes have reduced conversion of clopidogrel to its active metabolite and an increased risk for stent thrombosis after percutaneous coronary interventions. Pharmacogenomic implementation guidelines recommend that carriers of the CYP2C19 loss-of-function alleles *2 and *3 receive an alternative antiplatelet drug such as prasugrel or ticagrelor (Table 6).

**Table 6:** Recommendations for actionable pharmacogenomic markers based on CPIC guidelines.

Gene	Actionable SNP	MAF	Clinical Pharmacogenetics Implementation Consortium		
			Evidence level	Drug	Dosing guidelines
CYP2C9	rs1799853	0.015	1A	Warfarin	Pharmacogenetic algorithm-based dosing available on http://www.warfarindosing.org is used for patients with different combinations of CYP2C9 and VKORC1 genotypes.
	rs1057910	0.072	1A		
VKORC1	rs9923231	0.597	1A		
CYP2C19	rs4244285	0.342	1A	Clopidogrel, Amitriptyline	An alternative antiplatelet therapy to clopidogrel is recommended for CYP2C19 poor or intermediate metabolizers.
	rs4986893	0.025	1A		
	rs72552267	0.002	1A		An alternative drug to amitriptyline is recommended for CYP2C19 ultrarapid metabolizers.
	rs12248560	0.062	1A		
CYP2D6	rs16947	0.207	1A	Codeine, Amitriptyline, Nortiptyline	Alternate analgesics to codeine are recommended for CYP2D6 ultrarapid and poor metabolizers due to potential toxicity and lack of efficacy, respectively.
	rs1135840	0.617	1A		
	rs1080985	0.146	1A		Alternative drugs to amitriptyline and nortriptyline are recommended for CYP2D6 ultrarapid metabolizers and poor metabolizers.
	rs3892097	0.040	1A		
	rs1065852	0.411	1A		
	rs28371725	0.069	1A		
CYP3A5	rs776746	0.653	1A	Tacrolimus	Increasing the starting dose by 1.5 to 2 times is recommended for CYP3A5 intermediate or extensive metabolizers.

SNP, single-nucleotide polymorphism; MAF, minor allele frequency.

There was an overall high similarity in the allele frequencies among the Chinese and Malay populations. The Indian subjects exhibited greater genetic differences in specific variants of CYP2C9, CYP2C19, CYP2D6 and VKORC1 genes from the Chinese/Malay populations. One of the potential useful findings concerns the CYP2C9 and VKORC1 genes. We examined the genotype combinations of CYP2C9 and VKORC1 variants and noted that about 68.2% of Indians harboured the VKORC1 *1/*1 (GG) diplotype, implying that a substantial proportion of Indians require a higher starting dose of warfarin (Table 6) [10].

To our knowledge, this is the most comprehensive study of variation at the CYP2D6 locus in the Singapore population. A total of 17 alleles and duplication/multiplication were examined. CYP2D6*5 and *10 were previously shown to be prevalent in our population [29]. Our results are generally consistent with previous reports in that CYP2D6*2, *2A, *10 and *36 are found to be common and *5 and *41 to be relatively common in East Asians. We noted that CYP2D6*10 was mostly represented by *36 which are common in Asians. In this study, we tried to provide an exact copy number of functional CYP2D6 genes as accurate determination of the functional gene dosage is essential for detailed pharmacogenetic analysis of CYP2D6. The use of two assays targeting different regions of CYP2D6 serves to distinguish between *10 and *36 alleles [25]. In our population, EM was the most common phenotype for CYP2D6 followed by the IM. The prevalence of PM ranged from 0.7% to 3.4% and this phenotype was not observed in the Chinese subjects. We noted the highest proportion of UMs among the Chinese subjects (11%) followed by the Indian (5%) and Malay subjects (4.8%). Both PMs and UMs have an altered capacity to metabolize CYP2D6 substrates and could potentially benefit from genotyping. The typical substrates for CYP2D6 include codeine, some anti-depressants and antipsychotics. We predicted the response to codeine based on our results using CPIC guidelines [31]. Individuals who are UMs are at increased risk for toxicity due to increased formation of morphine following codeine administration (Table 6). By contrast, those carrying no functional alleles are at increased risk of insufficient pain relief due to lack of efficacy.

In conclusion, we described the potential use of a PGx panel in clinical testing. 506 subjects from our population were analysed on the panel and the knowledge of allele distribution and frequency is required to effectively translate pharmacogenetics to the clinics. Our study also showed that the vast majority of individuals (98%) have at least one actionable genotype even among the small range of drug gene rules. These findings have implications for warfarin, clopidogrel, codeine and anti-psychotics pharmacogenomics and provide impetus to incorporate PGx genotyping in our population.

## Supporting Information

S1 Fig Prediction of drug metabolizer phenotype. (DOCX)

S2 Fig Concordance between Taqman SNP genotyping assays and iPLEX PGx 68 Panel. (DOCX)

S3 Fig Quantitation of CYP2D6 gene copy numbers in 10 HapMap samples. (A) Quantitative real-time PCR results in CopyCaller software (in quadruplicates) with intron 2 and exon 9 assays in green and blue, respectively; (B) mass spectrometry analysis of CN based on intron 2 (in singleplex) and (C) absolute quantitaion based on exon 9 using ddPCR (in duplicates).
(TIF)
