# Supplementary material for: Analysis of Genetic Variation in CYP450 Genes for Clinical Implementation
Source: PLoS One. 2017 Jan 3;12(1):e0169233. doi: 10.1371/journal.pone.0169233 (PMC5207784; doi:10.1371/journal.pone.0169233)
Supplement: S1 Fig — (DOCX) [file pone.0169233.s001.docx]

| **Phenotype** | **Rule** |
| --- | --- |
| **A) CYP2C9 and VKORC1** |  |
| Extensive Metabolizer | Individuals carrying two normal function alleles |
| Intermediate Metabolizer | Individuals carrying :  i) one normal funciton allele plus one reduced function allele; ii) one normal function allele plus one loss-of-function allele; iii) two reduced function alleles |
| Poor Metabolizer | Individuals carrying: i) one reduced function allele plus one loss-of-function allele; ii) two loss-of-function alleles |
| **B) CYP2C19** |  |
| Ultra-rapid Metabolizer | Individuals carrying two increased function alleles |
| Extensive Metabolizer | Individuals carrying: i) two normal function alleles; ii) one normal function allele plus one increased function allele |
| Intermediate Metabolizer | Individuals carrying :  i) one normal function allele plus one reduced function allele; ii) one normal function allele plus one loss-of-function allele; iii) one loss-of-function allele plus one increased function allele; iv) two reduced function alleles |
| Poor Metabolizer | Individuals carrying: i) one reduced function allele plus one loss-of-function allele; ii) two loss-of-function alleles |
| **C) CYP2D6 with copy number variation** |  |
| Ultra-rapid Metabolizer | Individuals carrying three normal function alleles |
| Extensive Metabolizer to Ultra-rapid Metabolizer | Individuals carrying two normal function alleles plus one reduced function allele |
| Extensive Metabolizer | Individuals carrying: i) two normal function alleles; ii) two normal function alleles plus one loss-of-function allele; iii) one normal function allele plus two reduced function alleles |
| Intermediate Metabolizer to Extensive Metabolizer | Individuals carrying: i) one normal function allele plus one reduced function allele; ii) three reduced function alleles |
| Intermediate Metabolizer | Individuals carrying: i) one normal function allele plus one loss-of-function allele; ii) two reduced function alleles; iii) one normal function allele plus two or more loss-of-function alleles; iv) two reduced function alleles plus one loss-of-function allele |
| Poor Metabolizer to Intermediate Metabolizer | Individuals carrying: i) one reduced function allele plus one loss-of-function allele; ii) one reduced function allele plus two loss-of-function alleles |
| Poor Metabolizer | Individuals carrying two or more than two loss-of-function alleles |
| **D) CYP3A5** |  |
| Extensive Metabolizer | Individuals carrying two copies of *3 alleles |
| Intermediate Metabolizer | Individuals carrying :  i) two copies of *1 alleles; ii) one copy of *1 allele plus another copy of *3 allele |

**S1 Fig. Prediction of drug metabolizer phenotype.**
