# Supplementary material for: Analysis of Genetic Variation in CYP450 Genes for Clinical Implementation
Source: PLoS One. 2017 Jan 3;12(1):e0169233. doi: 10.1371/journal.pone.0169233 (PMC5207784; doi:10.1371/journal.pone.0169233)
Supplement: S2 Fig — (DOCX) [file pone.0169233.s002.docx]

| **Gene** | **Allele** | **dbSNP Number** | **TaqMan® SNP Genotyping Assays (Applied Biosystems)** | **iPLEX® PGx 68 Panel  (Agena Bioscience)** |
| --- | --- | --- | --- | --- |
| CYP2C9 | *2 | rs1799853 | √ | √ |
|  | *3 | rs1057910 | √ | √ |
|  | *4 | rs56165452 | √ | √ |
|  | *5 | rs28371686 | √ | √ |
|  | *6 | rs9332131 | √ | √ |
| CYP2C19 | *2 | rs4244285 | √ | √ |
|  | *3 | rs4986893 | √ | √ |
|  | *4 | rs28399504 | √ | √ |
|  | *5 | rs56337013 | √ | √ |
|  | *6 | rs72552267 | √ | √ |
|  | *7 | rs72558186 | √ | √ |
|  | *8 | rs41291556 | √ | √ |
|  | *9 | rs28399507 | √ |  |
|  | *10 | rs6413438 | √ |  |
|  | *17 | rs12248560 | √ | √ |
| CYP2D6 | *2 | rs16947 | √ | √ |
|  | *2 | rs1135840 | √ | √ |
|  | *2A | rs1080985 | √ |  |
|  | *3 | rs35742686 | √ | √ |
|  | *4 | rs3892097 | √ | √ |
|  | *6 | rs5030655 | √ | √ |
|  | *7 | rs5030867 | √ | √ |
|  | *8 | rs5030865 (1758G>T) | √ | √ |
|  | *9 | rs5030656 | √ | √ |
|  | *10 | rs1065852 | √ | √ |
|  | *14 | rs5030865 (1758G>A) | √ | √ |
|  | *17 | rs28371706 | √ | √ |
|  | *29 | rs59421388 | √ | √ |
|  | *35 | rs769258 | √ |  |
|  | *41 | rs28371725 | √ | √ |
| CYP3A5 | *3 | rs776746 | √ | √ |
| VKORC1 | *2 | rs9923231 | √ | √ |

**S2 Fig. Concordance between Taqman SNP genotyping assays and iPLEX PGx 68 Panel.**
