# Supplementary figures and images for: Analysis of Genetic Variation in CYP450 Genes for Clinical Implementation
Source: PLoS One. 2017 Jan 3;12(1):e0169233. doi: 10.1371/journal.pone.0169233 (PMC5207784; doi:10.1371/journal.pone.0169233)

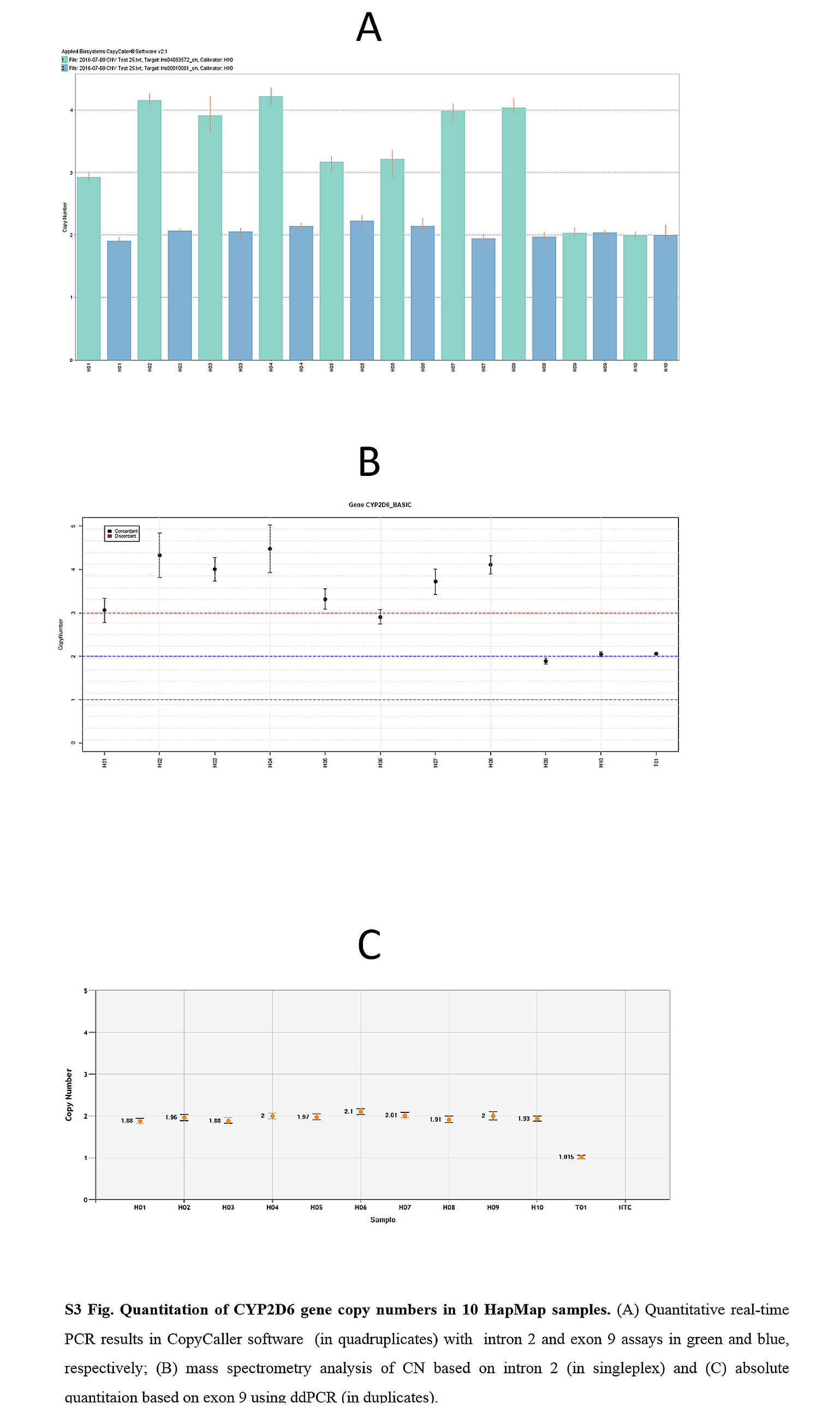

Supplement: S3 Fig — (A) Quantitative real-time PCR results in CopyCaller software (in quadruplicates) with intron 2 and exon 9 assays in green and blue, respectively; (B) mass spectrometry analysis of CN based on intron 2 (in singleplex) and (C) absolute quantitaion based on exon 9 using ddPCR (in duplicates). (TIF) [file pone.0169233.s003.tif]
